# Supplementary material for: Schistosome esophageal gland factor MEG-8.2 drives host cell lysis and interacts with host immune proteins
Source: bioRxiv. 2024 Nov 15:2024.11.15.623777. Preprint. [Version 1] doi: 10.1101/2024.11.15.623777 (PMC11601278; doi:10.1101/2024.11.15.623777)
Supplement: Supplement 5 [file NIHPP2024.11.15.623777v1-supplement-5.pdf]

bioRxiv preprint doi: https://doi.org/10.1101/2024.11.15.623777; this version posted November 15, 2024. The copyright holder for this preprint (which was not certified by peer review) is the author/funder, who has granted bioRxiv a license to display the preprint in perpetuity. It is made available under aCC-BY 4.0 International license.

A

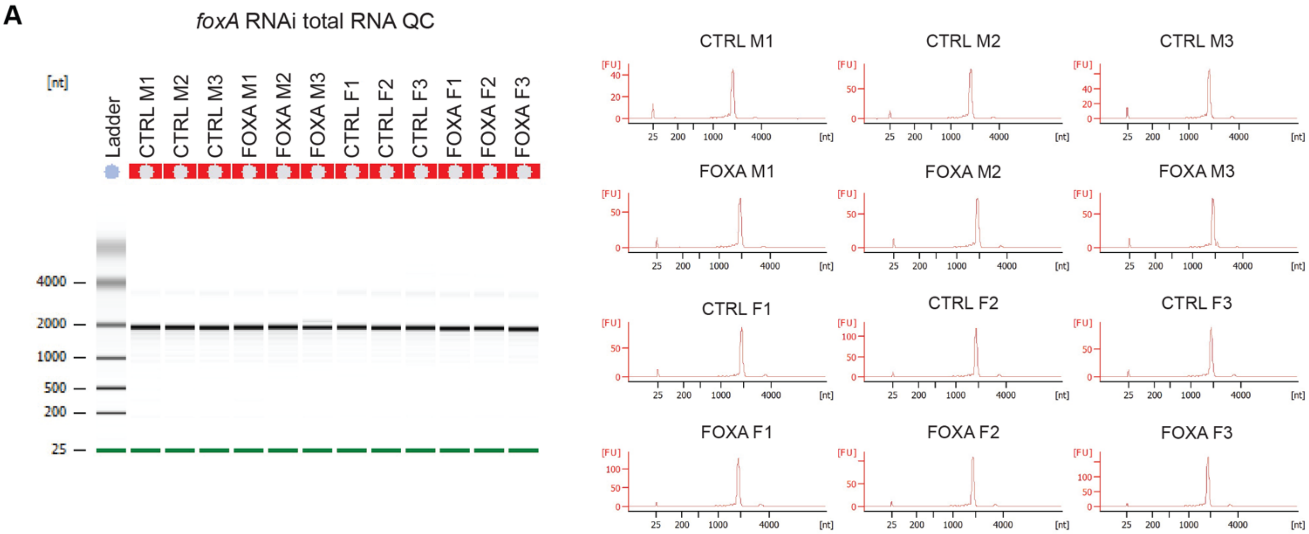

B

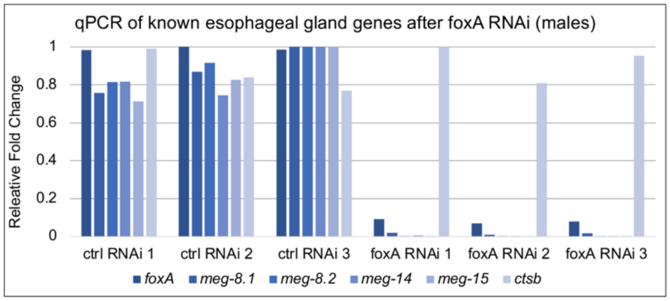

C

sample	ng/µL	A260/280	A260/230	A260	A280	total reads (paired)
CTRL M1	110.55	1.99	0.92	2.76	1.39	85853932
CTRL M2	133.8	1.93	1.41	3.34	1.73	93926256
CTRL M3	108.61	1.95	1.38	2.72	1.39	88773030
FOXA M1	124.06	1.94	1.3	3.1	1.6	82019926
FOXA M2	131.02	1.89	1.19	3.28	1.73	75907966
FOXA M3	115.53	1.87	1.15	2.89	1.54	99981462
CTRL F1	146.58	1.97	1.45	3.66	1.86	84216282
CTRL F2	160.23	2	1.63	4.01	2	100699262
CTRL F3	107.09	2.06	1.9	2.68	1.3	77949500
FOXA F1	166.78	2.04	1.64	4.17	2.05	83646150
FOXA F2	160	2.03	1.71	4	1.97	88082006
FOXA F3	204.84	2.08	1.87	5.12	2.47	74411150

Figure S1. Quality control of total RNA used in RNA-seq. (A) Bioanalyzer results of extracted total RNA samples. (B) qPCR of select known EG genes and non-EG genes (*ctsb*) in cDNA synthesized from the extracted RNA samples. The results show specific downregulation of EG genes in *foxA* knockdown. (C) Summary table of RNA concentration/quality and total reads sequenced for each sample.

bioRxiv preprint doi: https://doi.org/10.1101/2024.11.15.623777; this version posted November 15, 2024. The copyright holder for this preprint (which was not certified by peer review) is the author/funder, who has granted bioRxiv a license to display the preprint in perpetuity. It is made available under aCC-BY 4.0 International license.

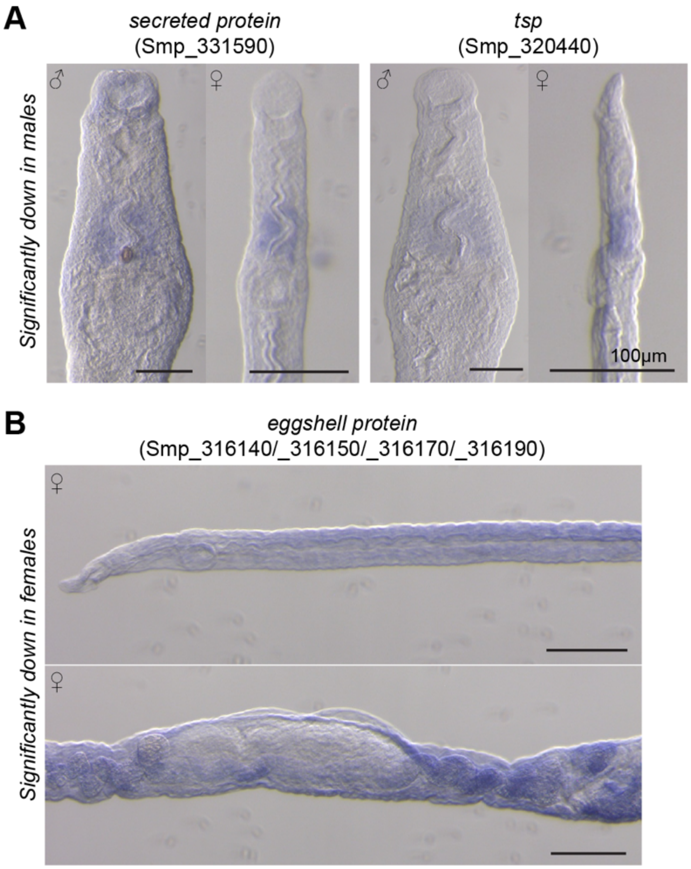

Figure S2. WISH of genes significantly downregulated in males (A) or females (B). (A) Both genes are significantly downregulated only in males but show slight enrichment in both males and females. (B) Eggshell protein downregulated in *foxA* RNAi females is not enriched in the EG but is likely enriched in the accessory reproductive tissues (e.g., vitellaria).

bioRxiv preprint doi: https://doi.org/10.1101/2024.11.15.623777; this version posted November 15, 2024. The copyright holder for this preprint (which was not certified by peer review) is the author/funder, who has granted bioRxiv a license to display the preprint in perpetuity. It is made available under aCC-BY 4.0 International license.

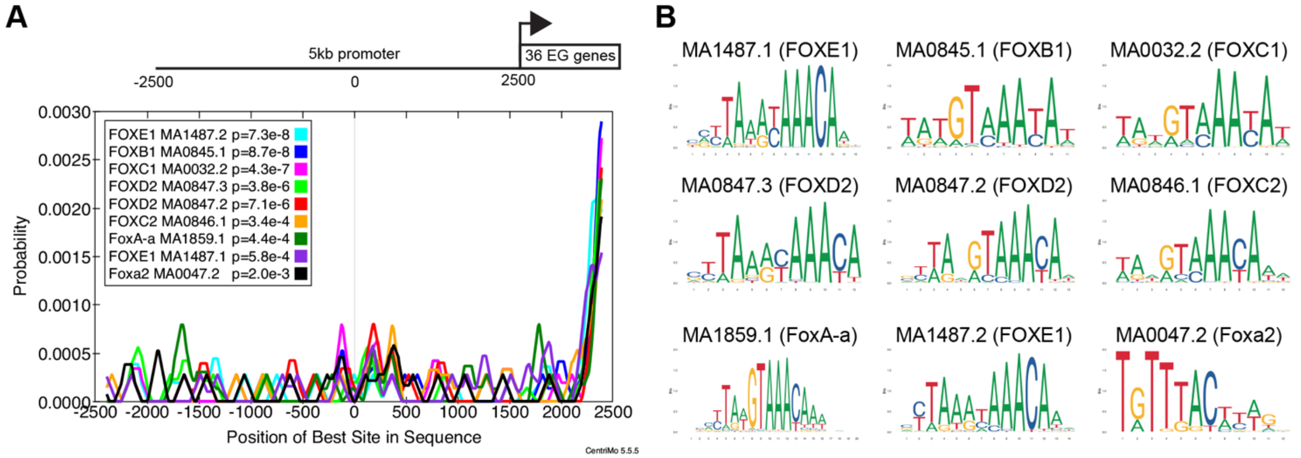

C

motif_id	motif_alt_id	consensus	E-value	adj_p-value	log_adj_p-value	bin_location	bin_width	total_width	sites_in_bin	total_sites	p_success	p-value	mult_tests
MA1487.2	FOXE1	VYTAAAYAAACAAH	1.80E-04	7.30E-08	-16.43	2376	179	4987	17	36	0.03589	5.90E-15	12437575
MA0845.1	FOXB1	WATGTAAATAT	2.10E-04	8.70E-08	-16.26	2376.5	181	4990	17	36	0.03627	7.00E-15	12447552
MA0032.2	FOXC1	WAWGTAAAYAW	1.00E-03	4.30E-07	-14.66	2415.5	103	4990	14	36	0.02064	3.40E-14	12447552
MA0847.3	FOXD2	SYTAARYAAACA	9.30E-03	3.80E-06	-12.47	2384.5	190	4989	16	36	0.03808	3.10E-13	12447552
MA0847.2	FOXD2	VYWAWGTAAACAW	1.70E-02	7.10E-06	-11.85	2376.5	179	4988	15	36	0.03589	5.70E-13	12437575
MA0846.1	FOXC2	WAWGTAAACAWW	8.30E-01	3.40E-04	-7.98	2362.5	154	4989	13	36	0.03087	2.70E-11	12447552
MA1859.1	FoxA-a	NBMYTAWGTAAACAAWNNNN	1.10E+00	4.40E-04	-7.72	2400	67	4981	10	36	0.01345	3.60E-11	12407668
MA1487.1	FOXE1	BVYTAAAYAAACAAH	1.40E+00	5.80E-04	-7.45	2331	268	4986	16	36	0.05375	4.70E-11	12427602
MA0047.2	Foxa2	TGTTTACWYWGB	4.90E+00	2.00E-03	-6.21	2376.5	178	4989	13	36	0.03568	1.60E-10	12447552

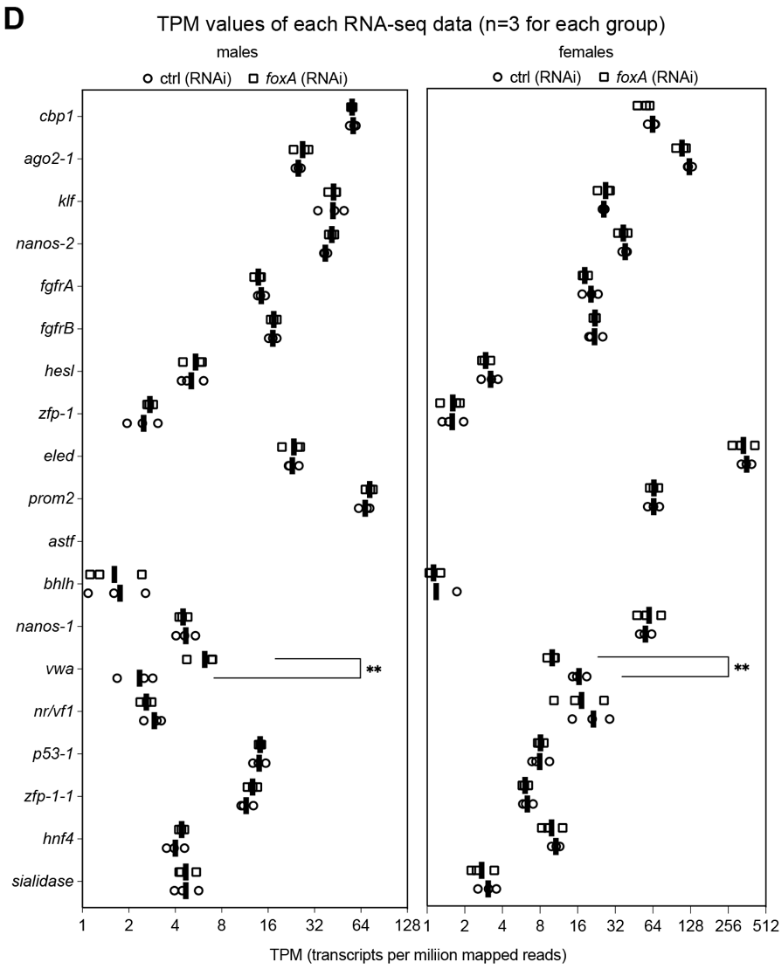

bioRxiv preprint doi: https://doi.org/10.1101/2024.11.15.623777; this version posted November 15, 2024. The copyright holder for this preprint (which was not certified by peer review) is the author/funder, who has granted bioRxiv a license to display the preprint in perpetuity. It is made available under aCC-BY 4.0 International license.

Figure S3. FoxA potentially regulates the EG genes' transcription directly but has little effect on other cell-type lineages. (A-C) CentriMo analysis of 5kb upstream sequences of 36 EG genes reveals putative forkhead transcription factor binding motifs on most promoters. (A) An overlay of the probability of each motif occurrence. (B) Enriched motifs. (C) A summary table of the location and the significance of each motif. (D) TPM values of cell-type progenitor markers in control and *foxA* RNAi males (left) and females (right). Except for *vwa*, a Mehlis gland marker, expression levels remain largely unchanged.

bioRxiv preprint doi: https://doi.org/10.1101/2024.11.15.623777; this version posted November 15, 2024. The copyright holder for this preprint (which was not certified by peer review) is the author/funder, who has granted bioRxiv a license to display the preprint in perpetuity. It is made available under aCC-BY 4.0 International license.

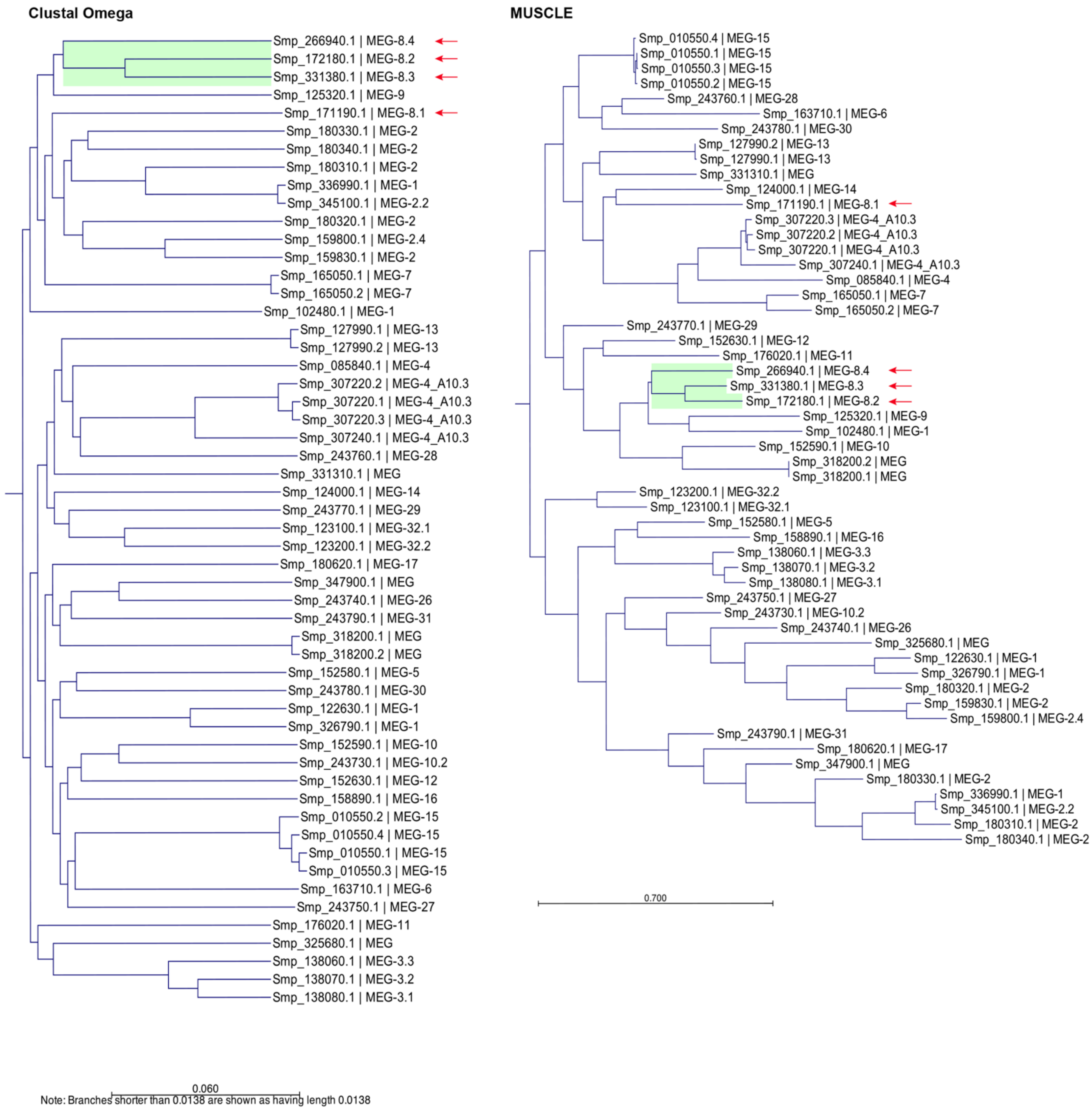

Figure S4. *S. mansoni* MEG family alignment. Left: Clustal Omega (v1.2.0); Right: MUSCLE (Algorithm: Neighbor Joining; Distance measure: Jukes-Cantor; Bootstrap: 100 replicates). Sm-MEG-8 family proteins are indicated with red arrows. Amino acid sequences for all of the proteins were derived from the *S. mansoni* genome (V10) available on WormBase ParaSite.

bioRxiv preprint doi: https://doi.org/10.1101/2024.11.15.623777; this version posted November 15, 2024. The copyright holder for this preprint (which was not certified by peer review) is the author/funder, who has granted bioRxiv a license to display the preprint in perpetuity. It is made available under aCC-BY 4.0 International license.

A MEG-8.2 BLAST against *Schistosomatidae* family

Hit	Description	Genome	Total score	Max score	Min E-value	Max %Identity
**Smp_172180.1 (Sm-MEG-8.2)**	transcript=Smp_172180.1 gene=Smp_172180	*Schistosoma mansoni (PRJEA36577)*	647	647	5.09E-87	100
SRDH2_89950.1	transcript=SRDH2_89950.1 gene=SRDH2_89950	*Schistosoma rodhaini (PRJEB44434 - tdSchRodh2.1)*	628	628	3.84E-84	97.86
SRDH1_92530.2	transcript=SRDH1_92530.2 gene=SRDH1_92530	*Schistosoma rodhaini (PRJEB44434 - tdSchRodh1.1)*	530	530	3.79E-69	80.82
SRDH1_92530.4	transcript=SRDH1_92530.4 gene=SRDH1_92530	*Schistosoma rodhaini (PRJEB44434 - tdSchRodh1.1)*	526	526	1.35E-68	82.39
SRDH1_92530.3	transcript=SRDH1_92530.3 gene=SRDH1_92530	*Schistosoma rodhaini (PRJEB44434 - tdSchRodh1.1)*	520	520	1.07E-67	72.6
SRDH1_92530.1	transcript=SRDH1_92530.1 gene=SRDH1_92530	*Schistosoma rodhaini (PRJEB44434 - tdSchRodh1.1)*	516	516	3.59E-67	73.94
SSPN1_84290.2	transcript=SSPN1_84290.2 gene=SSPN1_84290	*Schistosoma spindale (PRJEB44434 - tdSchSpin1.1)*	425	425	2.96E-53	66.91
SCUR1_86370.1	transcript=SCUR1_86370.1 gene=SCUR1_86370	*Schistosoma curassoni (PRJEB44434 - tdSchCurr1.1)*	415	415	8.04E-52	69.29
SMTH1_96980.1	transcript=SMTH1_96980.1 gene=SMTH1_96980	*Schistosoma mattheei (PRJEB44434 - tdSchMatt1.1)*	415	415	9.57E-52	67.86
SHAE1_89450.1	transcript=SHAE1_89450.1 gene=SHAE1_89450	*Schistosoma haematobium (PRJEB44434 - tdSchHaem1.1)*	411	411	3.68E-51	67.14
SBOV1_85840.1	transcript=SBOV1_85840.1 gene=SBOV1_85840	*Schistosoma bovis (PRJEB44434 - tdSchBovi1.1)*	410	410	5.70E-51	67.14
SBOV1_85840.1	transcript=SBOV1_85840.1 gene=SBOV1_85840	*Schistosoma bovis (PRJEB44434 - tdSchBovi1.1)*	410	410	5.70E-51	67.14
SBOV2_86550.1	transcript=SBOV2_86550.1 gene=SBOV2_86550	*Schistosoma bovis (PRJEB44434 - tdSchBovi2.1)*	409	409	5.83E-51	67.14
SINT2_85490.1	transcript=SINT2_85490.1 gene=SINT2_85490	*Schistosoma intercalatum (PRJEB44434 - tdSchInte2.1)*	408	408	9.12E-51	67.86
SINT1_85930.1	transcript=SINT1_85930.1 gene=SINT1_85930	*Schistosoma intercalatum (PRJEB44434 - tdSchInte1.1)*	408	408	9.12E-51	67.86
SGUI1_87820.1	transcript=SGUI1_87820.1 gene=SGUI1_87820	*Schistosoma guineensis (PRJEB44434 - tdSchGuin1.1)*	403	403	5.12E-50	68.66
SMRG1_88490.1	transcript=SMRG1_88490.1 gene=SMRG1_88490	*Schistosoma margrebowiei (PRJEB44434 - tdSchMarg1.1)*	393	393	1.90E-48	64.75
SMTH1_96980.2	transcript=SMTH1_96980.2 gene=SMTH1_96980	*Schistosoma mattheei (PRJEB44434 - tdSchMatt1.1)*	390	390	3.81E-48	62.86
SBOV2_86550.2	transcript=SBOV2_86550.2 gene=SBOV2_86550	*Schistosoma bovis (PRJEB44434 - tdSchBovi2.1)*	390	390	4.17E-48	62.86
SHAE1_89450.2	transcript=SHAE1_89450.2 gene=SHAE1_89450	*Schistosoma haematobium (PRJEB44434 - tdSchHaem1.1)*	387	387	1.09E-47	62.14
SINT2_85490.2	transcript=SINT2_85490.2 gene=SINT2_85490	*Schistosoma intercalatum (PRJEB44434 - tdSchInte2.1)*	380	380	1.12E-46	62.14
SINT1_85930.2	transcript=SINT1_85930.2 gene=SINT1_85930	*Schistosoma intercalatum (PRJEB44434 - tdSchInte1.1)*	380	380	1.12E-46	62.14
SSPN1_84290.1	transcript=SSPN1_84290.1 gene=SSPN1_84290	*Schistosoma spindale (PRJEB44434 - tdSchSpin1.1)*	376	376	6.02E-46	62.59
SMRG1_88490.2	transcript=SMRG1_88490.2 gene=SMRG1_88490	*Schistosoma margrebowiei (PRJEB44434 - tdSchMarg1.1)*	364	364	4.13E-44	61.87
STRK1_84990.1	transcript=STRK1_84990.1 gene=STRK1_84990	*Schistosoma turkestanicum (PRJEB44434 - tdSchTurk1.1)*	337	337	1.70E-39	47.43
EWB00_007522.2	transcript=EWB00_007522.2 gene=EWB00_007522	*Schistosoma japonicum (PRJNA520774 - HuSjv2)*	327	327	1.90E-38	55.4
GWHTBJUN006805	transcript=GWHTBJUN006805 gene=GWHGBJUN006806	*Schistosoma japonicum (PRJNA724792 - F4M4)*	323	323	6.95E-38	54.68
HAMR2_97370.4	transcript=HAMR2_97370.4 gene=HAMR2_97370	*Heterobilharzia americana (PRJEB44434 - tdHetHame2.1)*	251	251	8.45E-27	48.2
HAMR1_109440.1	transcript=HAMR1_109440.1 gene=HAMR1_109440	*Heterobilharzia americana (PRJEB44434 - tdHetHame1.1)*	248	248	1.43E-26	46.76
HAMR2_97370.1	transcript=HAMR2_97370.1 gene=HAMR2_97370	*Heterobilharzia americana (PRJEB44434 - tdHetHame2.1)*	247	247	4.50E-26	62.11
HAMR2_97370.3	transcript=HAMR2_97370.3 gene=HAMR2_97370	*Heterobilharzia americana (PRJEB44434 - tdHetHame2.1)*	246	246	7.72E-26	62.11
HAMR2_97370.2	transcript=HAMR2_97370.2 gene=HAMR2_97370	*Heterobilharzia americana (PRJEB44434 - tdHetHame2.1)*	245	245	9.50E-26	62.11
TREG1_121330.1	transcript=TREG1_121330.1 gene=TREG1_121330	*Trichobilharzia regenti (PRJEB44434 - tdTriRege1.1)*	199	199	6.32E-19	54.9
EWB00_007523	transcript=EWB00_007523 gene=EWB00_007523	*Schistosoma japonicum (PRJNA520774 - HuSjv2)*	196	196	7.98E-19	36.23
SRDH2_89940.1	transcript=SRDH2_89940.1 gene=SRDH2_89940	*Schistosoma rodhaini (PRJEB44434 - tdSchRodh2.1)*	188	188	8.27E-18	34.06
**Smp_331380.1 (Sm-MEG-8.3)**	transcript=Smp_331380.1 gene=Smp_331380	*Schistosoma mansoni (PRJEA36577)*	187	187	1.08E-17	34.06
SRDH1_92510.1	transcript=SRDH1_92510.1 gene=SRDH1_92510	*Schistosoma rodhaini (PRJEB44434 - tdSchRodh1.1)*	175	175	6.59E-16	31.88
SRDH1_92510.2	transcript=SRDH1_92510.2 gene=SRDH1_92510	*Schistosoma rodhaini (PRJEB44434 - tdSchRodh1.1)*	174	174	8.04E-16	50
SMTH1_96990.1	transcript=SMTH1_96990.1 gene=SMTH1_96990	*Schistosoma mattheei (PRJEB44434 - tdSchMatt1.1)*	169	169	3.94E-15	50
SCUR1_86360.1	transcript=SCUR1_86360.1 gene=SCUR1_86360	*Schistosoma curassoni (PRJEB44434 - tdSchCurr1.1)*	169	169	4.94E-15	33.33
SINT2_85500.1	transcript=SINT2_85500.1 gene=SINT2_85500	*Schistosoma intercalatum (PRJEB44434 - tdSchInte2.1)*	166	166	1.39E-14	55.36
SINT1_85940.1	transcript=SINT1_85940.1 gene=SINT1_85940	*Schistosoma intercalatum (PRJEB44434 - tdSchInte1.1)*	166	166	1.39E-14	55.36
SHAE1_89460.1	transcript=SHAE1_89460.1 gene=SHAE1_89460	*Schistosoma haematobium (PRJEB44434 - tdSchHaem1.1)*	166	166	1.39E-14	55.36
SGUI1_87830.1	transcript=SGUI1_87830.1 gene=SGUI1_87830	*Schistosoma guineensis (PRJEB44434 - tdSchGuin1.1)*	166	166	1.39E-14	55.36
SBOV2_86560.1	transcript=SBOV2_86560.1 gene=SBOV2_86560	*Schistosoma bovis (PRJEB44434 - tdSchBovi2.1)*	166	166	1.39E-14	55.36
SSPN1_84300.1	transcript=SSPN1_84300.1 gene=SSPN1_84300	*Schistosoma spindale (PRJEB44434 - tdSchSpin1.1)*	165	165	1.52E-14	46.97
SBOV1_85850.1	transcript=SBOV1_85850.1 gene=SBOV1_85850	*Schistosoma bovis (PRJEB44434 - tdSchBovi1.1)*	164	164	2.69E-14	55.36
SBOV1_85850.1	transcript=SBOV1_85850.1 gene=SBOV1_85850	*Schistosoma bovis (PRJEB44434 - tdSchBovi1.1)*	164	164	2.69E-14	55.36
SMRG1_88500.1	transcript=SMRG1_88500.1 gene=SMRG1_88500	*Schistosoma margrebowiei (PRJEB44434 - tdSchMarg1.1)*	158	158	2.00E-13	44.78
EWB00_007522.1	transcript=EWB00_007522.1 gene=EWB00_007522	*Schistosoma japonicum (PRJNA520774 - HuSjv2)*	131	131	2.54E-09	42.42
SSPN1_84300.3	transcript=SSPN1_84300.3 gene=SSPN1_84300	*Schistosoma spindale (PRJEB44434 - tdSchSpin1.1)*	106	106	6.59E-06	38.78
SRDH2_32390.1	transcript=SRDH2_32390.1 gene=SRDH2_32390	*Schistosoma rodhaini (PRJEB44434 - tdSchRodh2.1)*	100	100	3.55E-05	48.65
SRDH1_35720.1	transcript=SRDH1_35720.1 gene=SRDH1_35720	*Schistosoma rodhaini (PRJEB44434 - tdSchRodh1.1)*	102	102	5.52E-05	43.75
**Smp_266940.1 (Sm-MEG-8.4)**	transcript=Smp_266940.1 gene=Smp_266940	*Schistosoma mansoni (PRJEA36577)*	102	102	5.62E-05	42.86
SBOV1_33100.1	transcript=SBOV1_33100.1 gene=SBOV1_33100	*Schistosoma bovis (PRJEB44434 - tdSchBovi1.1)*	99	99	1.42E-04	51.52
SBOV1_33100.1	transcript=SBOV1_33100.1 gene=SBOV1_33100	*Schistosoma bovis (PRJEB44434 - tdSchBovi1.1)*	99	99	1.42E-04	51.52
SSPN1_84300.2	transcript=SSPN1_84300.2 gene=SSPN1_84300	*Schistosoma spindale (PRJEB44434 - tdSchSpin1.1)*	95	95	2.52E-04	37.5
SCUR1_34960.1	transcript=SCUR1_34960.1 gene=SCUR1_34960	*Schistosoma curassoni (PRJEB44434 - tdSchCurr1.1)*	95	95	3.77E-04	37.5
SSPN1_36110.1	transcript=SSPN1_36110.1 gene=SSPN1_36110	*Schistosoma spindale (PRJEB44434 - tdSchSpin1.1)*	93	93	9.93E-04	51.61
SMRG1_34880.1	transcript=SMRG1_34880.1 gene=SMRG1_34880	*Schistosoma margrebowiei (PRJEB44434 - tdSchMarg1.1)*	91	91	1.93E-03	50
SMTH1_38600.1	transcript=SMTH1_38600.1 gene=SMTH1_38600	*Schistosoma mattheei (PRJEB44434 - tdSchMatt1.1)*	90	90	2.47E-03	51.61
SINT2_33680.1	transcript=SINT2_33680.1 gene=SINT2_33680	*Schistosoma intercalatum (PRJEB44434 - tdSchInte2.1)*	91	91	2.49E-03	50
SINT1_34850.1	transcript=SINT1_34850.1 gene=SINT1_34850	*Schistosoma intercalatum (PRJEB44434 - tdSchInte1.1)*	91	91	2.49E-03	50
TREG1_57350.1	transcript=TREG1_57350.1 gene=TREG1_57350	*Trichobilharzia regenti (PRJEB44434 - tdTriRege1.1)*	87	87	4.86E-03	44.12
EWB00_008165.3	transcript=EWB00_008165.3 gene=EWB00_008165	*Schistosoma japonicum (PRJNA520774 - HuSjv2)*	87	87	7.59E-03	43.75
SHAE1_35740.1	transcript=SHAE1_35740.1 gene=SHAE1_35740	*Schistosoma haematobium (PRJEB44434 - tdSchHaem1.1)*	87	87	8.24E-03	48.39
**Smp_171190.1 (Sm-MEG-8.1)**	transcript=Smp_171190.1 gene=Smp_171190	*Schistosoma mansoni (PRJEA36577)*	89	89	9.30E-03	30.91
SRDH1_53940.1	transcript=SRDH1_53940.1 gene=SRDH1_53940	*Schistosoma rodhaini (PRJEB44434 - tdSchRodh1.1)*	89	89	9.43E-03	30.91

B

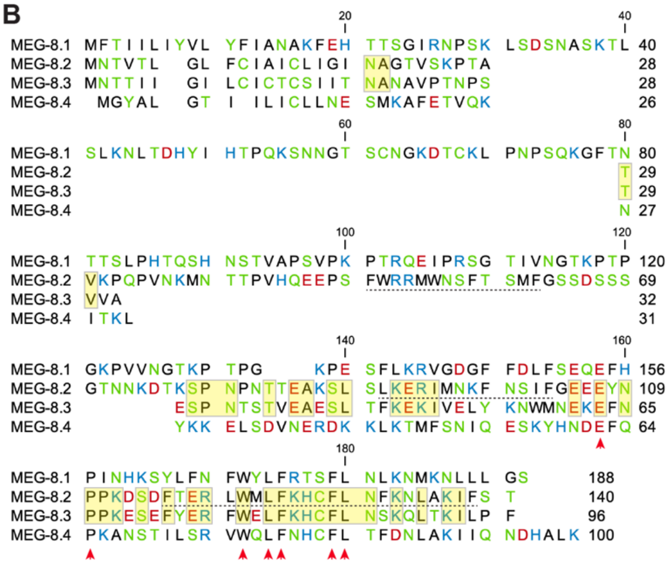

C

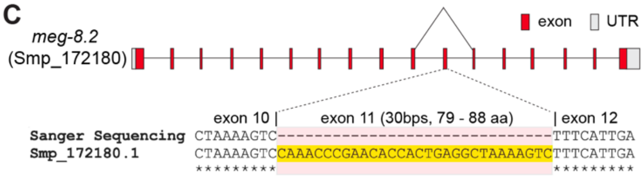

gene	cDNA source	sequence contains	number of clones	exons skipped
meg-8.2 (Smp_172180)	adult female	all exons	3	
	adult male	all exons	2	
	schistosomula (day 1)	all exons	3	
		skipped exon(s)	1	exon 11
	schistosomula (day 7)	all exons	1	

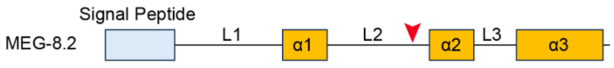

bioRxiv preprint doi: https://doi.org/10.1101/2024.11.15.623777; this version posted November 15, 2024. The copyright holder for this preprint (which was not certified by peer review) is the author/funder, who has granted bioRxiv a license to display the preprint in perpetuity. It is made available under aCC-BY 4.0 International license.

Figure S5. MEG-8 family alignment and a rare case of exon skipping. (A) BLAST of four Sm-MEG-8 proteins across the *Schistosomatidae* family show orthologs in other species. (B) Sm-MEG-8 alignment. The three predicted helices are indicated by a dotted underline. MEG-8.2 and MEG-8.3 share the most residues (yellow box). Residues shared across all four proteins are marked with a red arrowhead. (C) The regional sequence and location of skipped exon 11, which was found in one out of 10 sequenced clones.

bioRxiv preprint doi: https://doi.org/10.1101/2024.11.15.623777; this version posted November 15, 2024. The copyright holder for this preprint (which was not certified by peer review) is the author/funder, who has granted bioRxiv a license to display the preprint in perpetuity. It is made available under aCC-BY 4.0 International license.

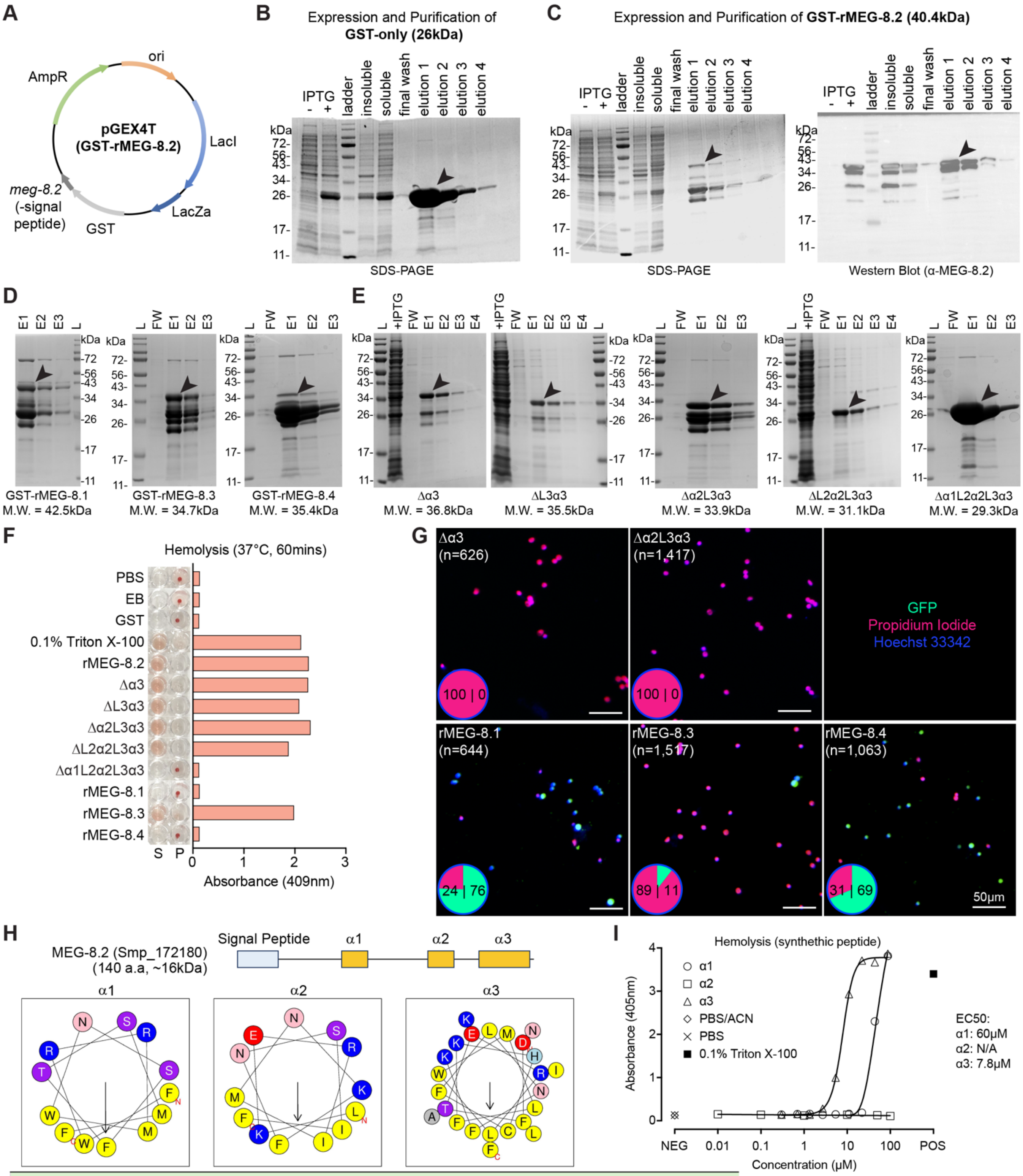

Helix Number	Sequence	Amino Acid Position	Length	Hydrophobicity	Hydrophobic Moment	Net charge (z)	Frequency of Polar Residues	Frequency of Non-Polar Residues
1	FWRRMWNSFTSMF	50 - 62	13 a.a.	0.761	0.778	2	0.46	0.54
2	LKERIMNKFNSIF	91 - 103	13 a.a.	0.403	0.594	2	0.54	0.46
3	DFTERLWMLFKHCFLNFKNLAKIF	115 - 138	24 a.a.	0.695	0.516	2	0.42	0.58

bioRxiv preprint doi: https://doi.org/10.1101/2024.11.15.623777; this version posted November 15, 2024. The copyright holder for this preprint (which was not certified by peer review) is the author/funder, who has granted bioRxiv a license to display the preprint in perpetuity. It is made available under aCC-BY 4.0 International license.

Figure S6. Cell lytic activity of recombinant MEG-8 proteins and mutants. (A) Schematic plasmid map of the bacterial expression vector used to express N-terminal GST-tagged MEG-8 proteins. GST-rMEG-8.2 is shown as an example. (B) SDS-PAGE of GST-only expression and purification steps. (C) Expression and purification of GST-rMEG-8.2. SDS-PAGE (left) shows the highest band corresponding to the expected molecular weight, while a few smaller-size proteins are observed. Western blot (right) using α-MEG-8.2 antibody confirms that these bands are positively labeled, suggesting that while rMEG-8.2 protein is produced, several degradation products are also in the purified mixture. (D) SDS-PAGE of other members of the Sm-MEG-8 family proteins. (E) SDS-PAGE of rMEG-8.2 truncation mutants. (B – D) Arrowheads indicate the expected band size. L: ladder; +IPTG: IPTG induced; FW: final wash; E1 – 4: elution 1 through 4. (F) Hemolysis assay using recombinantly purified MEG-8 proteins. Isolated peripheral blood was treated with indicated proteins for 60 minutes at 37°C. rMEG-8.2 containing α1 region retains the cell lytic activity, as well as rMEG-8.3, but not rMEG-8.1 or rMEG-8.4. EB; elution buffer only. (G) leukocyte lysis by rMEG-8.2 mutants (top) and other MEG-8 family proteins (bottom). GFP-expressing leukocytes were treated with each protein for 10 minutes at 37°C prior to adding PI and Hoechst33342. The pie chart on the lower left corner of each image indicates the viability. n: total number of cells counted. (H) HeliQuest analysis (*90*) of each of the predicted helices shows amphipathic properties, with α1 having the highest hydrophobicity and hydrophobic moment. (I) An independent second experiment of the dose curve of the synthetic peptides. EC50 values are largely in agreement with those shown in **Figure 4**.

bioRxiv preprint doi: https://doi.org/10.1101/2024.11.15.623777; this version posted November 15, 2024. The copyright holder for this preprint (which was not certified by peer review) is the author/funder, who has granted bioRxiv a license to display the preprint in perpetuity. It is made available under aCC-BY 4.0 International license.

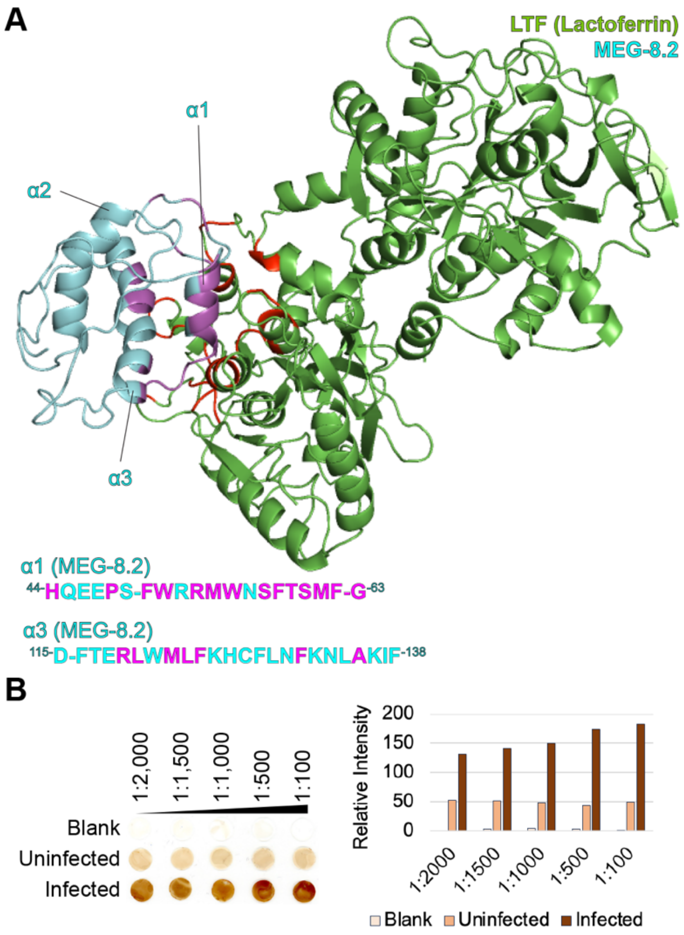

Figure S7. Modeling interaction between LTF and MEG-8.2. (A) MEG-8.2 residues with an interaction distance <4Å are highlighted in magenta. (B) Dot blot of MEG-8.2 in plasma lysate derived from infected and uninfected mice using a range of dilutions of anti-MEG-8.2 antibodies (see Methods).

Table S1. (separate file)

Differential expression analysis of *foxA* RNAi RNA-seq.

Table S2. (separate file)

LC-MS/MS analysis of rMEG-8.2 pull-down hits.

Table S3. (separate file)

bioRxiv preprint doi: https://doi.org/10.1101/2024.11.15.623777; this version posted November 15, 2024. The copyright holder for this preprint (which was not certified by peer review) is the author/funder, who has granted bioRxiv a license to display the preprint in perpetuity. It is made available under aCC-BY 4.0 International license.

List of oligonucleotides used in this study.

Table S4. (separate file)

Plasmid sequences for all constructs used for recombinant protein expression.
